# Editorial: Contemporary causes of acute myocarditis and pericarditis: diagnosis by advanced imaging techniques and therapeutic strategies

**DOI:** 10.3389/fcvm.2023.1211463

**Published:** 2023-05-16

**Authors:** Grigorios Korosoglou, Roohallah Alizadehsani, Sheikh Mohammed Shariful Islam, Andreas Rolf

**Affiliations:** ^1^ Department of Cardiology, Vascular Medicine and Pneumology, GRN Hospital Weinheim, Weinheim, Germany; ^2^ Cardiac Imaging Center Weinheim, Hector Foundation, Weinheim, German; ^3^ Institute for Intelligent Systems Research and Innovation (IISRI), Deakin University, Geelong, VIC, Australia; ^4^ Faculty of Health, Deakin University, Burwood, VIC, Australia; ^5^ Department of Cardiology, Campus Kerckhoff of the Justus Liebig, University Giessen, Bad Nauheim, Germany; ^6^ German Centre for Cardiovascular Research (DZHK), Partner Site RheinMain, Bad Nauheim, Germany

**Keywords:** cardiac magnetic resonance (CMR), late gadolinium enhancement (LGE), multimodal imaging, tissue characterization, COVID-19, vaccination, perimyocarditis

**Editorial on the Research Topic** Contemporary causes of acute myocarditis and pericarditis: diagnosis by advanced imaging techniques and therapeutic strategies

The incidence of myocarditis was estimated to be ∼22/100,000 patients annually before the beginning of the COVID-19 pandemic in 2020 (1, 2). A large proportion of patients with acute myocarditis may develop dilated cardiomyopathy with symptomatic heart failure, which can also be associated with future adverse outcomes and high mortality rates (3).

The clinical symptoms of myocarditis are angina, dyspnea, and fatigue. In addition, malignant arrhythmias causing sudden cardiac death are feared, especially in younger individuals (4). Since the beginning of the pandemic, the number of patients affected by myocarditis has increased, both due to SARS-COVID infections and due to vaccination-induced myocarditis, the latter occurring more commonly in young male individuals who received mRNA-related vaccines (5, 6).

Based on contemporary guidelines, cardiac magnetic resonance (CMR) plays a central role in the diagnosis of myocarditis, however, endomyocardial biopsy should only be used for select patients due to its invasive character. CMR is an established non-invasive imaging technique, aiding the diagnosis of myocarditis by late gadolinium enhancement (LGE), which provides evidence of focal fibrosis or oedema due to inflammation and by T1&T2 mapping, which provides evidence of diffuse fibrosis of the extracellular space and myocardial edema, respectively. In this regard, CMR does not only aid diagnostic classification and risk stratification of such patients but may also influence important clinical decisions, such as tailoring heart failure therapy, indication for ICD or wearable CD, duration of immobilization, and time point for a return to work or play.

The role of CMR and myocardial strain for the detection of classical myocarditis was investigated by Motevalli et al. in 133 patients. The myocardial strain was assessed by feature tracking imaging. In particular, the results indicated that the global longitudinal strain (GLS) was a strong predictor of major adverse cardiac events during the follow-up period.

Numerous studies have investigated patients with COVID-19 vaccine associated myocarditis and pericarditis. Jahnke et al. describe a series of four young male individuals (18–42 years.) who presented with signs of myocarditis in temporal association with SARS-CoV-2 vaccines and abnormal CMR findings. In another study, Vago et al. investigated 16 young male patients (22 ± 7 years.), who presented with vaccine related myocarditis. CMR depicted myocardial oedema by T2 weighted and mapping images, and myocardial necrosis by LGE, which decreased or disappeared during follow-up. Interestingly, mostly individuals with predisposing immunologic factors including previous myocarditis were affected, exhibiting increased T-cell response compared to controls. Similar CMR findings were demonstrated by Kravchenko et al. who investigated vaccine associated myocarditis patients with CMR at baseline and at ∼6 months of follow-up. Myocardial oedema disappeared during follow-up, areas of LGE were smaller compared to baseline, and clinical symptoms were resolved in most patients. Additionally, Evertz et al. investigated a series of 10 young male patients with COVID-19 mRNA vaccine-associated myocarditis and an age-matched group of patients suffering from classical viral myocarditis. Using CMR, they found that vaccine-related myocarditis shows similar patterns of myocardial oedema and LGE compared to regular viral myocarditis. Furthermore, Ochs et al. described an interesting series of 5 patients, who presented with clinical symptoms related to myocarditis but showed isolated pericarditis by CMR diagnostic work-up, which included LGE, T1, and T2 mapping. The patients were older (median of 55 years., IQR = 43–76) than the patients with vaccine related myocarditis described in other series, but the underlying pathophysiologic mechanisms require further evaluation in future studies.

Three further studies of this Research Topic focus on patients with COVID-19 related myocarditis, partly comparing CMR related findings to patients with classical viral or with SARS-CoV-2 vaccine associated myocarditis. Tanacli et al. performed baseline and part follow-up CMR examinations in 32 patients with persistent cardiac symptoms after COVID-19 infection. Interestingly, 10 (31%) of the patients with COVID-19 showed evidence of myocardial injury by CMR, while the number was reduced to only 3 (9%), considering the updated Lake Louise criteria. In addition, none of the COVID-19 patients exhibited histologic findings of acute or chronic inflammation by endomyocardial biopsy. In the same direction, Groschel et al. investigated 34 patients with subacute and 63 with post-COVID-19 infection by CMR. In addition, 44 patients with vaccine-related myocarditis were included in their study. The investigators found that patients after COVID-19 infection exhibited more focal fibrosis, primarily having a non-ischemic subepicardial pattern, compared to patients with vaccine-related myocarditis. However, LV and RV-function and diameters were within the normal range both in post-COVID and vaccine-related myocarditis cases. In addition, Zhang et al. conducted an autopsy study on the hearts of 26 deceased patients who had been hospitalized in intensive care units due to COVID-19 infection in Wuhan, China. Active myocarditis was present in 4 (15.4%) patients, who also exhibited higher interleukin values, higher CRP values at admission, and higher troponin values during hospitalization, compared to the remaining 22 patients. Finally, Xu et al. provide an interesting overview of studies related to COVID-19 associated cardiac disorders, including myocarditis and heart failure. This article demonstrates how initial research in this area was often focused on pneumonia. Then, with the spread of the disease, the infection was found to trigger an inflammatory response that resulted in cardiac injury. Therefore, the keywords myocarditis, heart failure, and cardiac troponins have become increasingly prominent.

In conclusion, the role of CMR as a central tool for the diagnosis of classical viral myocarditis has been reestablished within the COVID pandemic era. Several studies and case series reported in this article collection demonstrate how the ability to diagnose it can enable better support and clinical management in patients with COVID-19 and vaccination-related myocarditis and pericarditis. With the end of the ongoing pandemic hopefully approaching, future studies need to focus on longer-term follow-up examinations and care for these patients.
